# A Microfluidic System for Studying the Effects of Disturbed Flow on Endothelial Cells

**DOI:** 10.3389/fbioe.2019.00081

**Published:** 2019-04-17

**Authors:** Francisco Tovar-Lopez, Peter Thurgood, Christopher Gilliam, Ngan Nguyen, Elena Pirogova, Khashayar Khoshmanesh, Sara Baratchi

**Affiliations:** ^1^School of Engineering, RMIT University, Melbourne, VIC, Australia; ^2^School of Health and Biomedical Sciences, RMIT University, Bundoora, VIC, Australia

**Keywords:** microfluidics, endothelial cells (EC), disturbed flow, shear stress, actin stress fiber

## Abstract

Arterial endothelium experience physical stress associated with blood flow and play a central role in maintaining vascular integrity and homeostasis in response to hemodynamic forces. Blood flow within vessels is generally laminar and streamlined. However, abrupt changes in the vessel geometry due to branching, sharp turns or stenosis can disturb the laminar blood flow, causing secondary flows in the form of vortices. Such disturbed flow patterns activate pro-inflammatory phenotypes in endothelial cells, damaging the endothelial layer and can lead to atherosclerosis and thrombosis. Here, we report a microfluidic system with integrated ridge-shaped obstacles for generating controllable disturbed flow patterns. This system is used to study the effect of disturbed flow on the cytoskeleton remodeling and nuclear shape and size of cultured human aortic endothelial cells. Our results demonstrate that the generated disturbed flow changes the orientation angle of actin stress fibers and reduces the nuclear size while increases the nuclear circularity.

## Introduction

Endothelial cells, lining the inner surface of blood vessels, are in direct contact with the flowing blood, and their response to physiological as well as pathological flow dynamics affects vascular health (Chiu and Chien, 2011; Baratchi et al., 2017a). Endothelial cells experience various flow patterns and hemodynamic forces across the vascular system. Endothelial cells lining along the straight segments of the arterial trees experience a high-shear laminar flow essential for their physiological functions such as flow-mediated dilation (Chiu and Chien, 2011). In contrary, the endothelial cells lining along the arterial branches and curvatures experience a low-shear disturbed flow due to the presence of secondary flows in the form of vortices, which causes endothelial dysfunction and atherosclerosis (Caro et al., 1969, 1971; Bharadvaj et al., 1982; Suo et al., 2008).

Flow-induced alignment of the endothelial cells has been reported previously (Alenghat and Ingber, 2002; Wang et al., 2013; Yoshino et al., 2017). Recent advances in endothelial mechanobiology have also demonstrated that different classes of mechanoreceptors control the physiological function of endothelial cells, including cytoskeleton remodeling, gene expression, cell viability index and calcium homeostasis that could consequently control myogenic tone (Ingber, 2006; Chatzizisis et al., 2007; Shemesh et al., 2015). Shear-induced activation of various proteins such as RAC1 (Tzima et al., 2002) and Syndecan 4 (Baeyens et al., 2014) as well as enzymes such as RhoGTPases (Kroon et al., 2017) have also been reported to control the alignment and cytoskeleton remodeling of endothelial cells.

A variety of models have been developed to study the effect of disturbed flow on endothelial cells. These include *in vivo* models through surgical intervention (Katoh et al., 2007; Harding et al., 2018), *ex vivo* models using the endothelium at naturally occurring disturbed flow regions of the vessel (Katoh et al., 2007), and *in vitro* models using emerging microfluidic systems (Estrada et al., 2011). Among these models, microfluidic systems offer unprecedented advantages such as reduced cost and complexity of experiments, decreased volume of reagents (Katt et al., 2016; Ho et al., 2018; Yaman et al., 2018), and importantly provide predictable and controllable disturbed flow patterns based on the geometric specifications of the system (Rezvan et al., 2011; Balaguru et al., 2016). Despite these advantages, the majority of existing microfluidic systems generate localized disturbed flow patterns and thus are not suitable for studying the gene and protein expression of cultured cells under disturbed flow (Rezvan et al., 2011; Balaguru et al., 2016).

Here, we developed a microfluidic system with an array of ridge-shaped obstacles patterned along its entire surface. This allows for the generation of vortices and, in turn, low-shear disturbed flow regions in the cavities located between the successive ridges. This feature is used to quantify the effect of disturbed flow on the actin cytoskeleton remodeling, nucleus shape and size of cultured human aortic endothelial cells.

## Materials and Methods

### Fabrication of 2D Parallel-Plate Flow Chambers

**Figure 1:**
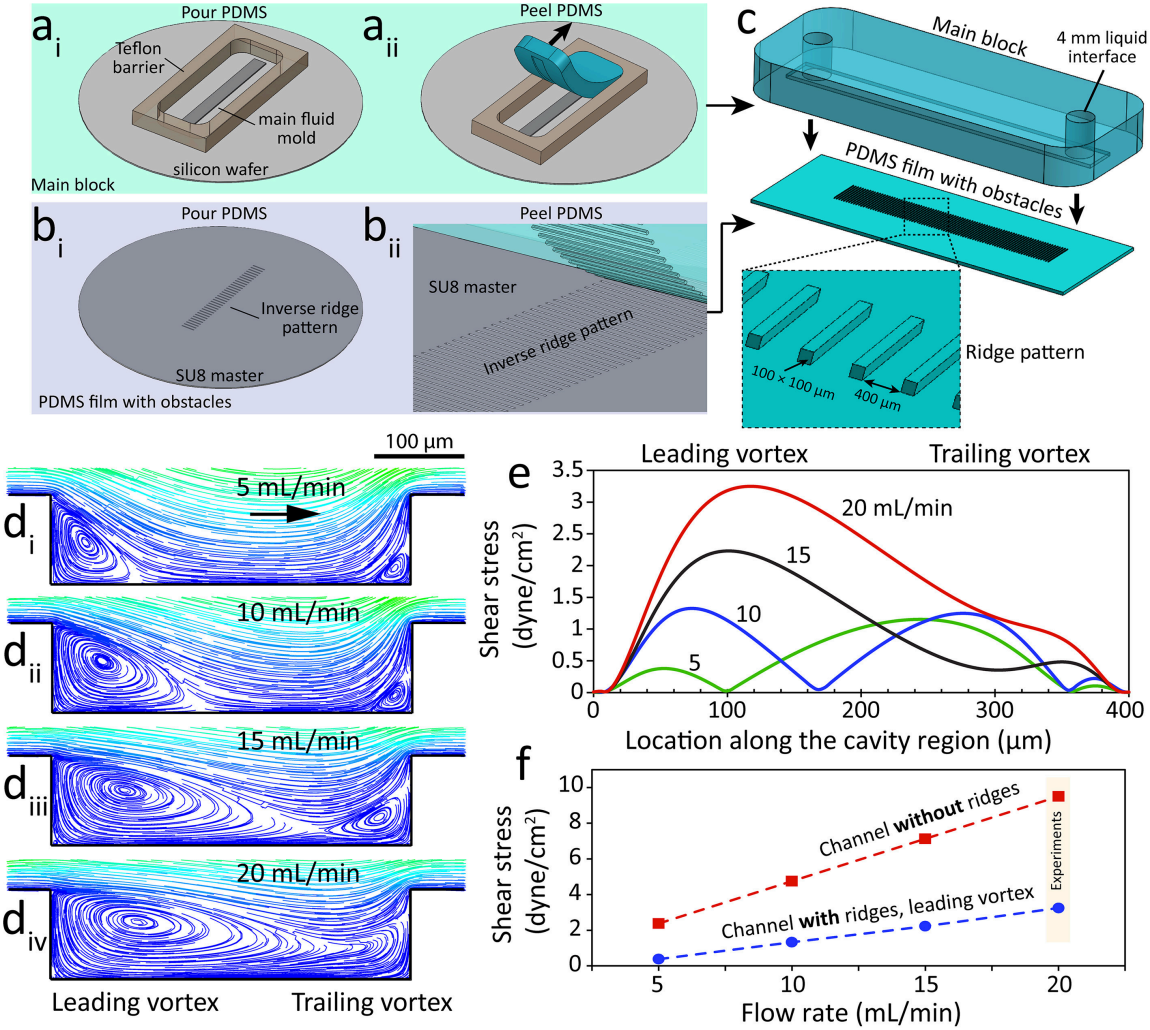
A microfluidic system with ridge-shaped obstacles for culturing HAECs under disturbed flow. **(a**_**i−ii**_**)** The process of fabricating the main block involving a microfluidic channel with a rectangular cross-section of 5 mm × 600 μm forming the side and top walls of the system, **(b**_**i−ii**_**)** The process of fabricating the PDMS substrate with ridge-shaped obstacles patterned on its surface forming the bottom surface of the system, **(c)** The process of assembling the main block and the PDMS substrate with ridge obstacles, with the inset showing the zoomed-in PDMS substrate, **(d**_**i−iv**_**)** Flow streamlines at various flow rates of the cell culture medium applied through the microfluidic system, showing the formation and expansion of two vortices at the cavity region located between the neighboring ridges, **(e)** Variation of flow shear stress along the cavity region, **(f)** Variation of shear stress magnitude against the flow rate of the cell culture medium for the channel with ridges (disturbed flow) and the control channel without ridges (laminar flow).

Ridged flow chambers were fabricated in two parts: (i) the main PDMS block consisting of a 500 μm tall channel and (ii) a PDMS film consisting of an array of 100 μm tall ridges. The main PDMS block was fabricated using a silicon wafer mold diced into a 5 mm × 50 mm × 500 μm section and adhered on to a 4-inch silicon wafer and enclosed with a 6 mm tall Teflon barrier (22 × 60 × 6 mm) (Figure 1a_i_). PDMS (Sylgard® 184, 10:1 w/w base to curing agent) was poured into the Teflon barrier and cured at 80°C for 30 min. The PDMS block was then peeled off the mold and inlet, and outlet holes were punched using a 4 mm biopsy punch (Harris Unicore) (Figure 1a_ii_).

The PDMS film with ridges was fabricated using a mold consisting of an array of rectangular grooves (100 μm × 100 μm × 5 mm) separated by 400 μm gaps. The mold was patterned in SU-8 3050 photoresist (MicroChem) using a high-resolution chrome mask (Figure 1b_i_). PDMS was then poured onto the mold and spun at 100 rpm for 30 s before curing at 85°C for 5 min. The resulting PDMS film was then peeled off the mold revealing the array of rectangular obstacles. The PDMS film was cut to 22 × 60 mm prior to the assembly of the two parts (Figure 1b_ii_). The PDMS main block and PDMS film with ridges were manually aligned and clamped together using a PMMA clamp resulting in a ridged flow chamber with rectangular obstacles (Figure 1c). A third mold, similar to the PDMS film mold, but without ridges was used to fabricate flat PDMS films for use as the control.

### Computational Fluid Dynamics (CFD)

CFD simulations were conducted to predict the formation of vortices along the ridged microfluidic channel. This involved solving the differential equations governing the balance of mass and momentum, also known as Navier-Stokes equations. Simulations were performed using ANSYS Fluent software (ANSYS Inc.). Simulations were conducted in 3D and under steady-state conditions, considering the cell culture medium as an incompressible and Newtonian liquid. Flow was considered laminar due to its low Reynolds number. Boundary conditions included desired flow rates at the inlet, ambient pressure at the outlet and no-slip at the walls. The density and dynamic viscosity of the cell culture medium at 37°C were considered as 998 kg/m^3^ and 7 × 10^−4^ Pa.s, respectively.

### Cell Culture and *in-vitro* Generation of Laminar vs. Disturbed Flow

HAECs (Lonza) at early passages of 2 to 5 were used in this study. HAECs were cultured inside the microfluidic channels at the density of 5 × 10^6^ cell/mL to produce a confluent layer of endothelial cells within 24 h. The volume of liquid inside the channel was ~100 μL. The channels were pre-coated with 10 μg/mL MaxGel human extracellular matrix extracts (Sigma) according to the manufacturer's specification.

Flow experiments were carried out for 16 h at 37°C in a humidified atmosphere with 5% CO_2_. The flow was applied through the channels using a peristaltic pump (OINA QP6 LAB High Accuracy) at flow rates of 20 mL/min and 6 mL/min.

### Immunocytochemistry and Confocal Microscopy

Cells were fixed with 4% paraformaldehyde and permeabilised with 0.2% Triton X-100 in phosphate-buffered saline (PBS). Nonspecific binding was blocked with 2% goat serum or 5% bovine serum albumin (Sigma). F-actin was stained with Atto 565-phalloidin (94072, Sigma-Aldrich, dilution 1/500) and nuclei were stained with DAPI (Sigma-Aldrich). Image acquisition was obtained using a Nikon A1 confocal scanning microscope, as described before (Baratchi et al., 2016).

### Image Processing and Analysis of Stress Fibers

The orientation of the cells and stress fibers was determined using an automated image processing algorithm written in MATLAB, as detailed elsewhere (Karlon et al., 1999; Kaunas et al., 2005; Ranade et al., 2014). Briefly, the algorithm computed the intensity gradients of the image in the horizontal and vertical directions by convolving the image with a spatial gradient filter. By treating these gradients as components of a vector field, the algorithm computed pixel-by-pixel magnitude and direction information. Local dominant orientations were then determined for each pixel by constructing a histogram of orientations from the magnitude and direction information and choosing the orientation with the largest value. Each histogram was constructed using a small subregion of 20 × 20 pixels, centered around the pixel of interest, and evaluating their deviation from a set of angles ranging from −89 to 90 degrees relative to the horizontal. For more details, we refer the reader to Kroon et al. (2017). Statistical significance was assessed with a global Watson's U2 test, and statistics were computed using the circular statistic toolbox (Berens, 2009).

### Quantification of Nuclear Shape and Size

Image processing and calculations of nuclear shape and size were performed using NIS element software (Nikon Instruments Inc.). For statistical analysis, one-way ANOVA was performed using Prism 7.02 (GraphPad software) and *P* < 0.05 was considered significant.

## Results and Discussions

### Characterization of Disturbed Flow

Microfluidic structures with the ridged or grooved patterns induce secondary flows in the form of vortices. This feature has been utilized for enhancing passive mixing of liquids (Stroock et al., 2002) as well as cell-based studies involving sorting (Yan et al., 2017), capturing (Manbachi et al., 2008; Stott et al., 2010; Hsiao et al., 2016; Wang et al., 2017) and forced tethering (Choi et al., 2014) of target cells.

An extensive set of numerical simulations were performed to analyze the disturbed flow patterns inside our microfluidic channel with integrated ridge-shaped obstacles. Our simulations indicated the formation of two vortices along the two corners of the cavity located between the successive ridges, which are referred to as “leading” and “trailing” vortices (Figure 1d). Our experiments confirmed the generation of “leading” and “trailing” vortices at the two corners of the cavity (Supplementary Video 1). Experiments were performed at a flow rate of 5 mL/min using a suspension of 1 μm polystyrene particles to facilitate the visualization of vortices. Increasing the flow rate of the cell culture medium led to the expansion of the vortices. In this manner, the two vortices occupied almost the entire cavity region at flow rates higher than 15 mL/min. Further increase of the flow rate led to merging of “leading” and “trailing” vortices.

The magnitude and distribution of the wall shear stress were governed by the configuration of the vortices (Figure 1e). Numerical simulations revealed the existence of three localized shear peaks at 5 mL/min, corresponding to the “leading” vortex, vortex-free zone and “trailing” vortex. A similar pattern was observed at 10 mL/min. Two localized shear peaks were obtained at 15 mL/min due to the diminishing of the vortex-free zone. In comparison, only one localized shear peak was obtained at 20 mL/min due to the merging of “leading” and “trailing” vortices.

The shear stress induced by the “leading” vortex increased linearly with respect to the flow rate (Figure 1f). A similar trend was obtained for the control channel with no ridges (Figure 1f). Based on these results, the cellular experiments were performed at a flow rate of 20 mL/min to ensure (i) the endothelial cells cultured inside the “channel with ridges” experience a low shear stress under homogenous disturbed flow conditions while (ii) the endothelial cells cultured inside the “channel without ridges” experience physiological shear stress under laminar flow conditions (Chiu and Chien, 2011).

### Disturbed Flow Affects the Orientation of Actin Stress Fibers and Nuclear Shape Change

Flow shear stress controls different endothelial phenotypic characteristics, including cell morphology, cytoskeleton remodeling and gene expression (Caro et al., 1969, 1971; Bharadvaj et al., 1982; Suo et al., 2008). Cytoskeleton proteins play important roles in maintaining the shape and integrity of the cells as well as transduction of shear stress from the luminal surface of endothelial cells to the cytosol (Loufrani and Henrion, 2008). Actin stress fibers consist of bundles of 10–30 actin filaments that are held together by actin crosslinking protein known as α-Actinin (Pellegrin and Mellor, 2007). Stress fibers are very important transducers of shear stress and transmit the stress to various intracellular locations (Franke et al., 1984; Wechezak et al., 1985).

HAECs were cultured overnight inside the channels with ridge-shaped obstacles (disturbed flow), without ridges (laminar flow) as well as inside Petri dishes (static condition). The flow rate of the medium was set to 20 mL/min. Under this condition, the cells cultured under disturbed flow experienced low shear stress with a maximum of 3 dyne/cm^2^, whereas the cells cultured under laminar flow experienced physiological shear stress with a magnitude of ~10 dyne/cm^2^, as detailed in the previous section.

**Figure 2:**
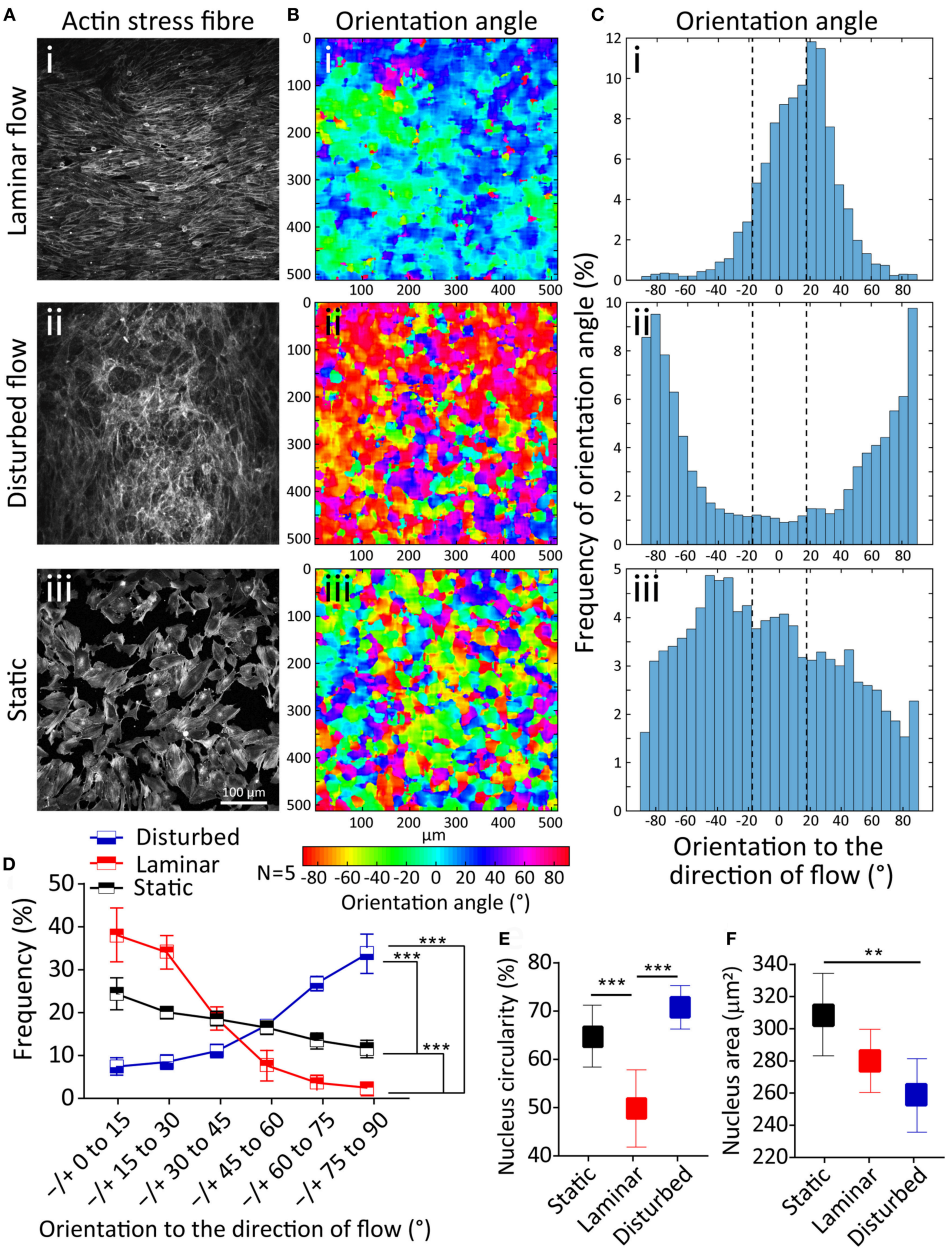
Effect of disturbed flow on cytoskeleton remodeling, nucleus circularity and nucleus size of HAECs. **(A**_**i−iii**_**)** A confluent layer of HAECs cultured under laminar flow, disturbed flow and static condition, following which the actin cytoskeleton was labeled with Atto 565-phalloidin after fixation. **(B**_**i−iii**_**)** The contour of the orientation angle of stress fibers, and **(C**_**i−iii**_**)** Histogram of the frequency of the orientation angle of stress fibers, **(D)** Summary graph of the frequency of the orientation angle of stress fibers cultured under laminar flow, disturbed flow and static condition. The graph is obtained from five independent experiments. ***Indicates *P* < 0.001, **(E)** Circularity, and **(F)** area of the nucleus of HAECs. Data are representative of five independent experiments, and error bars shown in **(D-F)** represent 95% confidence interval. 70 nuclei have been analyzed for each group. **Indicates *P* < 0.01 and ***P < 0.001.

First, we studied the orientation of stress fibers developed under the laminar flow, disturbed flow, and static condition. Under the laminar flow, HAECs stress fibers were highly orientated to the direction of flow (*R*^2^ = 0.93), with 71.59 ± 3.2% of stress fibers having an orientation angle of 0–30°. In contrary, in the presence of the disturbed flow, the majority of stress fibers were oriented perpendicular to the direction of flow (*R*^2^ = 0.92), with 59.74 ± 1.9% of stress fibers having an orientation angle of 60–90° (Figure 2A). In comparison, under static condition, the orientation of stress fibers did not follow any specific trend (Figure 2A). This trend can be clearly seen in the contours of the stress fiber orientation angle, obtained by our automated image processing algorithm. The trend shows a transition from green-blue colors (corresponding to the orientation angles in the range of ±30°) toward red-purple colors (corresponding to orientation angles outside the range of ±70°) (Figure 2B). This trend can also be seen in the histograms of the stress fiber orientation angle, which clearly shows the significant changes of stress fiber orientation under disturbed flow condition (Figure 2C). Consistent results were obtained in five independent experiments (Figure 2D). Next, we conducted a control experiment, by applying a flow rate of 6 mL/min through a straight microfluidic channel without ridges, to induce a shear stress of 3 dyne/cm^2^. Our experiments indicated that stress fibers were aligned perpendicular to the direction of the flow, similar to what we observed under the disturbed flow conditions inside the channel with ridges. This observation suggests that the response of HAECs to the disturbed flow is dependent on the magnitude of shear stress, rather than the flow pattern of the small vortices formed between the ridges (Supplementary 1).

Alignment of endothelial cells to the direction of flow has been observed previously (Alenghat and Ingber, 2002; Wang et al., 2013; Yoshino et al., 2017). It has been demonstrated that endothelial cells have the threshold of 10 dyne/cm^2^ to align to the direction of flow which is similar to what we have also observed in our preliminary studies (Wang et al., 2013; Ostrowski et al., 2014). Further, porcine valvular endothelial cells have been demonstrated to orient perpendicular to the direction of flow in response to the shear stress of 20 dyne/cm^2^ in comparison to the aortic endothelial cells that are aligned in parallel to the flow direction at the same shear stress level (Butcher et al., 2004; Baratchi et al., 2017b; Nguyen et al., 2018). Morphology of endothelial cells (shape and size) and actin cytoskeleton is reported to be different at the branch point of the aorta where the blood flow is disturbed compared to the regions of the aorta where blood flow is laminar (Katoh et al., 2007). This can be attributed to the different responses of endothelial cells to different shear levels, as reported in our work.

The nucleus in eukaryotic cells is the site of transcriptional regulation and receives the mechanical stress that is transmitted via the cytoskeleton. Both intra and extracellular forces affect the nuclear shape and structure (Dahl et al., 2008). To evaluate this phenomenon, we compared the nuclear shape index and size of HAECs under the laminar flow, disturbed flow and static conditions. Disturbed flow significantly increased the nucleus circularity index of HAECs compared to the laminar flow (*P* < 0.001) and static condition (*P* < 0.001) (Figure 2E). In comparison, the nuclear size had a significantly smaller area under the disturbed flow compared to the laminar flow (*P* < 0.01) and static condition (*P* < 0.01) (Figure 2F). The observed nuclear shape change in the presence of different flow dynamics can be attributed to the change in the mechanically induced signaling or macromolecular conformational changes related to change in gene expression, in the presence of different flow dynamics (Davies, 2009).

## Conclusions

Here, we reported the microfluidic-based *in vitro* model for generating the disturbed flow that mimics the pathological flow patterns of arterial branch point and curvatures. The observed flow disturbance is due to the formation of vortices along the ridges. We showed that the expansion of vortices and the magnitude of the wall shear stress can be tuned by varying the flow rate of the cell culture medium through the system. At the flow rate of 20 mL/min, the vortices filled the entire cavity region between the neighboring ridges, inducing a maximum wall shear stress of 3 dyne/cm^2^ along the bottom surface of the channel where endothelial cells were cultured. To demonstrate the capability and efficacy of this model, we studied the effect of the disturbed flow on endothelial cytoskeleton remodeling and stress fiber formation as well as its nuclear shape and size. We used a MATLAB code to quantify the orientation of actin stress fibers under the laminar flow, disturbed flow and static condition.

Our results indicated that the generated disturbed flow affects the morphology and cytoskeleton remodeling of HAECs. Under the laminar flow, endothelial cells were aligned to the direction of flow and formed actin stress fibers, whereas the low-shear disturbed flow caused endothelial cells to orient perpendicular to the direction of flow. Furthermore, the HACEs exhibited a significantly higher nucleus circularity index and smaller nuclear size in the presence of disturbed flow.

These observations demonstrated the suitability of the presented microfluidic system for studying the effect of disturbed flow on the biology of endothelial cells, providing unique opportunities for evaluating the effect of the disturbed flow on the expression and function of different mechanoreceptors in endothelial cells.

## Author Contributions

FT-L fabricated the microfluidic device. PT conducted experiments and analyzed the results. CG wrote the MATLAB code for automated processing of images. NN conducted experiments. EP wrote the manuscript. KK performed numerical simulations and wrote the manuscript. SB designed the study, performed experiments, wrote the manuscript and supervised the work.

### Conflict of Interest Statement

The authors declare that the research was conducted in the absence of any commercial or financial relationships that could be construed as a potential conflict of interest.
