# Supplementary material for: A Microfluidic System for Studying the Effects of Disturbed Flow on Endothelial Cells
Source: Front Bioeng Biotechnol. 2019 Apr 17;7:81. doi: 10.3389/fbioe.2019.00081 (PMC6499196; doi:10.3389/fbioe.2019.00081)
Supplement: Supplementary file 1 [file Data_Sheet_1.PDF]

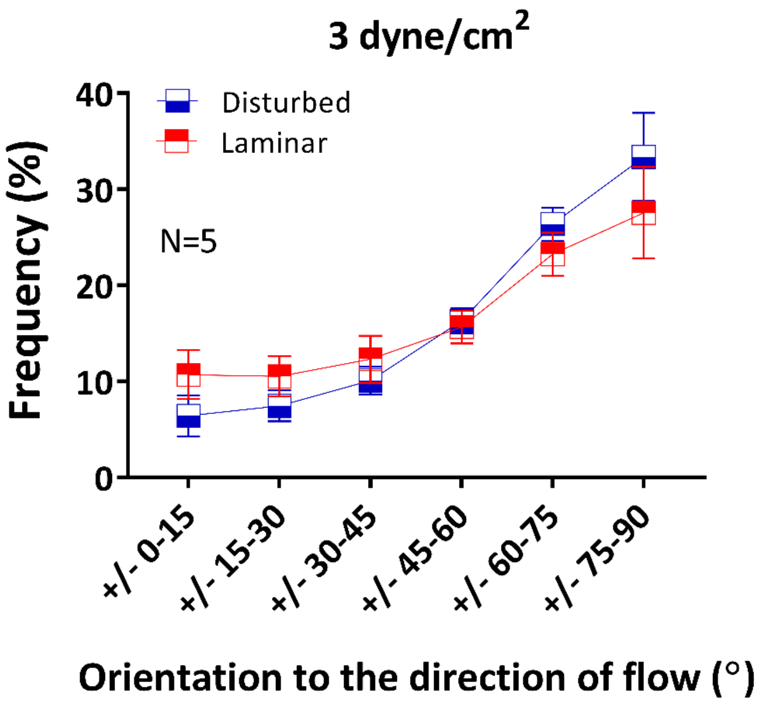

Supplementary 1. Summary graph showing that there is no difference on the frequency of the orientation angle of stress fibres cultured under shear stress of 3 dyne/cm^2 induced by laminar flow (using a channel without ridges at 6 mL/min) and disturbed flow (using a channel with ridged-shaped structues at 20 mL/min). Error bars represent 95% confidence interval.
